# Syndromic and non-syndromic etiologies causing neonatal hypocalcemic seizures

**DOI:** 10.3389/fendo.2022.998675

**Published:** 2022-11-10

**Authors:** Yi-Chieh Huang, Yin-Chi Chao, Inn-Chi Lee

**Affiliations:** ^1^ Division of Pediatric Neurology, Department of Pediatrics, Chung Shan Medical University Hospital, Taichung, Taiwan; ^2^ Institute of Medicine, School of Medicine, Chung Shan Medical University, Taichung, Taiwan

**Keywords:** Alström syndrome, newborns, hypocalcemic, syndromic, gene

## Abstract

**Background:**

The diagnosis of neonatal hypocalcemic seizures (HS) in newborns is made based on clinical signs and serum calcium level. Their etiology is broad and diverse, and timely detection and initiation of treatment is essential.

**Methods:**

We retrospectively reviewed 1029 patients admitted to the neonatal intensive care unit. Neonatal HS were diagnosed in 16 patients, and we compared etiologies and clinical outcomes, including clinical seizures and neurodevelopment at least over 1 year old.

**Results:**

The etiologies can be broadly categorized into 5 syndromic and 11 non-syndromic neonatal HS. Syndromic neonatal HS included 3 Digeorge syndrome, 1 Kleefstra syndrome and 1 Alström syndrome. Non-syndromic neonatal HS included 8 vitamin D deficiency, 1 hypoparathyroidism, and 2 hypoxic-ischemic encephalopathy. Patients with syndromic neonatal HS were found to have worse clinical outcomes than those with nonsyndromic HS. In eight patients with vitamin D deficiency, neurodevelopment was normal. Five of five patients (100%) with syndromic HS used two or more antiseizure drugs. However, among patients with non-syndromic neonatal HS, only one of 11 (9.1%) used more than one drug (*p =* 0.001).

**Conclusion:**

This finding highlighted that syndromic hypocalcemic seizures in newborns have worse neurodevelopmental outcomes and are more often difficult to manage, and would benefit from a genetic diagnostic approach.

## Introduction

Newborn infants are found to be at a higher risk of experiencing brain disorders, encephalopathies, and seizures. The neonatal hypocalcemic seizures (HS) are a group of disorders with diverse and complex etiology. The diagnosis of neonatal HS is currently based only on patients’ clinical presentation and laboratory examination (1–3). Early diagnosis and rapid treatment are essential in patients with neonatal seizures (4). Identification of etiology and immediate initialization of effective therapy could help clinicians to manage the severity of additional comorbidities.

Calcium is the one of most important ion in neonatal nerve conduction. Hypocalcemia, either symptomatic or asymptomatic, is a common metabolic disorder in newborns. Symptomatic hypocalcemia manifests in the form of common clinical symptoms, such as jitteriness, muscle jerking, and seizures. After birth, the serum level of calcium in newborns is affected by several factors, such as parathyroid hormone secretion, dietary intake, renal reabsorption function, skeletal calcium storage, and vitamin D level. In a healthy 2-day term infant, calcium levels decrease to a physiological nadir of 7.5–8.5 mg/dL (5). Hypocalcemia with PTH elevation can be caused by increased phosphate load, vitamin D deficiency, vitamin D metabolism defect, renal dysfunction, hypomagnesemia, genetic mutations resulting in end-organ resistance to PTH, or critical illness (6–9). Hypocalcemic seizures are can be classified as early onset (manifestation before 5 days of life) or late onset (manifestation after 5 days of life). Early-onset seizures can commonly occur in both preterm and term newborns. Late-onset seizures have a more complex and varied etiology. Syndromic neonatal HS include DiGeorge syndrome and other genetic disorders (often not identified). Non-syndromic neonatal HS include parathyroid gland disorders and acquired causes such as vitamin D deficiency. However, the etiology remains unknown in some cases. A previous retrospective review summarized clinical and laboratory characteristics as well as outcomes of neonates presenting with transient late-onset hypocalcemia and reported that the majority of patients had low vitamin D levels (6). All patients responded to magnesium and calcium supplementation and the administration of calcitriol and low phosphate formula (6). The outcome of vitamin D deficiency is relatively benign; however, in syndromic HS, it is unclear and noteworthy.

The use of whole exome sequencing (WES) to diagnose neonatal HS has been rarely reported. The application of WES to other unknown causes of seizures can help further elucidate the etiologies, initialize early treatment, predict outcomes, prevent a potential diagnostic odyssey, and greatly improve our understanding of the pathophysiology at the molecular level (10, 11). However, WES is rarely utilized in the diagnosis of neonatal HS and the genotype-phenotype correlation remains complicated and not fully understood.

In this study, we discuss a case-series of hypocalcemic seizures with various etiologies in newborns. Using the advanced technique of WES, rare etiologies found in neonatal hypocalcemic seizures can be better understood and the outcomes can be predicted. To evaluate the etiologies need more comprehensive care for those neonatal HS.

## Materials and methods

### Patients

From 2017 to 2020, 16 patients with neonatal HS, who met the hypocalcemia criteria (serum calcium level < 8 mg/dL [2.0 mmol/L] and ionized calcium level < 4.0 mg/dL [1.0 mmol/L]) were included in the study (6, 12, 13). Intact parathyroid hormone (iPTH) and vitamin D levels were measured for each patient if possible. All patients with HS were administered intravenous calcium, with or without anti-seizure drugs, and then switched to oral medication. Refractory hypocalcemia was defined as a duration of hypocalcemia of ≥ 7 days despite calcium supplementation. Additionally, genetic examination was performed in patients with comorbidities, including congenital heart diseases and other organ or endocrine disorders. Genetic testing comprised chromosome and whole exome sequencing (WES); WES was performed if the results of the chromosome study were negative. We arranged EEG monitoring for all patients with seizures. To clarify whether HS was caused by hypocalcemia or associated complex comorbidities, we defined acute seizures due to hypocalcemia as the cessation of seizures after normalization of serum calcium levels. We reviewed patient charts retrospectively and analyzed clinical presentations and etiologies that led to neonatal HS. Levels of 25-hydroxyvitamin D ≤ 25 ng/mL (62.4 nmol/L) were defined as abnormally lowered (6). Patients with neonatal hypoxic-ischemic encephalopathy (HIE) were as follows: 1. placental abruption, cord prolapse, or fetal distress; 2. Apgar score ≤ 5 in the 10th minute after birth; 3. need for ventilation beyond the tenth minute of life; and 4. acidosis (arterial pH of <7.20 or base deficit of ≥ 10 mmol/L) and must present with clinical encephalopathy by lethargy, stupor, or coma after birth. Patients with congenital abnormalities, chromosomal anomalies, premature birth (< 36 weeks), or syndromes that involved brain dysgenesis were excluded. Neonatal HIE was classified according to clinical Sarnat staging as I (mild), II (moderate), and III (severe) (14–16).

Neurodevelopmental outcomes after 1 year of age were evaluated using Bayley Scales of Infant and Toddler Development (Bayley-III) (17, 18). With regard to Bayley-III scores, cognitive scores were interpreted as: normal, ≥ 85 points; mild, ≥ 70 and < 85 points; moderate, ≥ 55 and < 70 points; and severe, < 55 points (17, 18).

### Genetic and whole exome sequencing analysis

A genomic DNA purification kit (Gentra Puregene Buccal Cell Kit, Qiagen Taiwan, Taipei City) was used to extract genomic DNA from peripheral whole blood sample taken from each patient after their legal guardian or parents signed the informed consent from. DNA for WES was extracted from peripheral blood taken from index cases and their parents and was stored at − 80°C. Variant calling was performed using the recommended best practices with GATK v1.0.5506 software (Broad Institute). Variant annotation and prioritization was performed using a well-developed pipeline: wANNOVAR (19). A few commonly used functional annotations, such as different types of gene annotations, alternative allele frequency in the 1000 Genomes Project, conserved element annotation, dbSNP annotation, deleteriousness prediction scores for nonsynonymous variants, ClinVar variant annotation and genome-wide association study variant annotation, were included in the results (20). All the identified variants were further confirmed by Sanger sequencing. The corresponding genes and individual phenotypes (clinical, laboratory, and imaging data) were evaluated according to the OMIM database. Segregation analysis was carried out to select *de novo* or compound heterozygous variants. Identified variants were classified as “pathogenic,” “likely pathogenic,” or “uncertain significance” according to the American College of Medical Genetics standards and guidelines (21).

Ethical approval was obtained from Chung Shan Medical University Hospital’s Internal Review Board (IRB #: CS14003) and the study was performed in accordance with relevant guidelines.

### Statistical analysis

Statistical differences between different groups were analyzed using an independent T-test using Statistical Package for the Social Sciences 14.0 statistical software (SPSS Institute, Chicago, IL, USA). Significant differences were evaluated using an independent T-test or a chi-square (χ^2^) test. In the event that sample sizes were relatively small, Fisher’s exact was performed. Statistical significance was set at *p* < 0.05.

## Results

### Demographic data

**Table 1** summarizes different etiologies and clinical presentations of 16 patients with HS (6 males, and 10 females). All 16 patients with neonatal HS were term (gestational age ≥ 37 weeks at the time of birth). Among the 16 patients, the follow-up duration was 3.9 ± 1.0 years of mean age (ranged: 2.5–6.2 years). Age of those who underwent Bayley-III test was 2.2 ± 0.6 years of mean age (ranged: 1.5–3.1 years). Vitamin D deficiency was found in eight patients, syndromic neonatal HS was found in five, two had hypoxic-ischemic encephalopathies (HIE) stage I, and one had hypoparathyroidism. The analysis of the number of antiepileptic medications prescribed for syndromic neonatal HS revealed that more anti-seizure drugs were taken by patients in the syndromic group than those in the non-syndromic group. Five out of five (100%) patients with syndromic neonatal HS used two or more drugs, whereas only one patient with non-syndromic neonatal HS out of 11(9.1%) used more than one drug (*p =* 0.001). This indicated that seizures due to syndromic etiologies, particularly those with genetic origins, were more frequent and required more antiepileptic drugs to control seizures.

**Table 1:** Clinical presentations and etiologies of hypocalcemic seizures in 16 newborns.

Case number		Patient 1		Patient 2	Patient 3		Patient 4	Patient 5		Patient 6		Patient 7		Patient 8		Patient 9	
**Etiology**		Digeorge syndrome		Vit. D deficiency	Digeorge syndrome		Kleefstra syndrome	Vit D deficiency		Vit D deficiency		HIE (stage I)		Vit. D deficiency		Vit. D deficiency	
**Phenotype**		HS, interrupted aortic arch, VSD		Later-onset HS	HS, imperforated anus, vascular ring		HS, ambiguous genitalia, scrotal bifida with hypospadias, penoscrotal transposition, congenital heart disease with DORV, anterior malalignment VSD, ASD, and PS	Later-onset HS, hyperphosphatemia		Later-onset HS, hyperphosphatemia		HS, hyperphosphatemia		HS, hyperphosphatemia		HS, hyperphosphatemia	
**Lowest calcium level**		6.2 mg/dl		6.9 mg/dl	6.3 mg/dl		6.9 mg/ dL	4.3 mg/dl		5.6 mg/dl		7.0 mg/dl		6.6 mg/dl		5.7 mg/dl	
**Duration of hypocalcemia (days)**		15		5 days	10		4 days	10 days		8 days		3 days		14 days		7 days	
**Response to calcium^％^**		Refractory		Easy	Refractory		Easy	Refractory		Refractory y		Easy		Refractory		Easy	
**Age of first Sz**		Day 5		Day 6	Day 5		Day 4	Day 5		Day 8		Day 3		Day 2		Day 8	
**Age of Dx**		6 months		Day 16	3 months		3 months	Day 15		Day 14		Day 4		Day 15		Day 14	
**Inheritance**		AD		Nil	AD		AD	Nil		Nil		Nil		Nil		Nil	
**Genotype**		22q11.2 deletion			22q11.2 deletion		Ring chromosome 9										
**Sex**		Female		Female	Female		Male	Female		Female		Female		Male		Male	
**Sz types**		Myoclonic		Focal	Generalized tonic		Focal	Focal		Myoclonic		Focal		Focal		Myoclonic	
**History of drugs control**		IV PB, PHT, then oral TOP, pyridoxine		Calcitriol	IV PB, PHT, Calcitriol		IV PB, PHT and oral calcitriol for HS. LEV, pyridoxine for seizures after 2 years and 3 months.	PB, Calcitriol		PB, Calcitriol		PB, calcitriol		Calcitriol, calcium chloride		PB, calcitriol calcium chloride	
**Abnormal MRI**		Mild ventriculomegaly		NA	Mild ventriculomegaly		Corpus callosum hypoplasia	NA		NA		Mild ventriculomegaly.		NA		NA	
**Neurodevelopmental outcomes**		Severe delay		Unremarkable	Moderate delay		Mild delay	Unremarkable		Unremarkable		Unremarkable		Unremarkable		Unremarkable	
Case number		Patient 10		Patient 11			Patient 12				Patient 13		Patient 14		Patient 15		Patient 16	
**Phenotype**		HS		HS,hyperphosphatemia, hypomagnesemia, dilated cardiomyopathy, PPHN with respiratory failure			HS, hypoglycemia, growth hormone deficiency, insulin resistance			HS, high iPTH, VSD		Later-onset HS		HS, Interrupted aortic arch		Later-onset HS	
**Lowest Ca level**		6.9 mg/dl		4.9 mg/dl			6.3 mg/dl			4.5 mg/dl		5.6 mg/dl		6.6 mg/dL		6.6 mg/dl	
**Duration of hypocalcemia (days)**		5 days		8 days			8 days			18 d		8 days		7day		4 days	
**Response to Ca supply^％^**		Easy		Refractory			Refractory			Refractory		Refractory		Refractory		Easy	
**Age of first Sz**		Day 3		Day 6			Day 8			Day 2		Day 6		Day 5		Day 6	
**Age of Dx**		Day 5		4 months			4 months			3 months		Day 15		4 months		Day 14	
**Inheritance**		Nil		AR			AR			Nil		Nil		AD		Nil	
**Genotype**				Two alleles *ALMS1* c.12118-65C>T and c.12118-65C>T (in cis)			Compound heterozygous *ALMS1*.c.10079C>G (p.A3360G)/ c.10323G>C (p.K3441N)							22q11.2 deletion			
**Gender**		Male		Female			Female			Male		Female		Male		Female	
**Sz types**		Myoclonic		Focal			Focal			Generalized tonic		Focal		Focal clonic seizures		Focal seizure	
**History of drug control**		Calcium carbonate, PB, calcitriol		IV PB, PHT, calcitriol, calcium chloride, calcium Carbonate			PB, PHT			IV PB, oral PB		Calcitriol		IV PB, PHT then oral PB, calcitriol		Calcitriol	
**Abnormal MRI**		NA		NA			Mild ventriculomegaly			Mild ventriculomegaly		NA		NA		NA	
**Neurodevelopmental outcomes**		Unremarkable		Unremarkable			Unremarkable			Mild delay		Unremarkable		Moderate delay		Unremarkable	

^%^Refractory hypocalcemia was defined as a hypocalcemia duration of ≥ 7 days despite calcium supply. All patients received calcium supply by intravenous calcium after the diagnosis of HS.

Sz, seizure; Dx, diagnosis; HS, hypocalcemic seizures; VSD, ventricular septal defect; DORV, double outlet right ventricle; ASD, atrial septal defect; PS, pulmonary stenosis; PDA, patent ductus arteriosus; PPHN, persistent pulmonary hypertension of newborn; AD, autosomal dominant; AR, autosomal recessive; IV, intravenous; PHT, phenytoin; OXC, oxcarbazepine; TOP, topiramate; PB, phenobarbital; LEV, levetiracetam; OXC, oxcarbazepine; SAB, vigabatrin; MRI, magnetic resonance imaging; iPTH, intact parathyroid hormone; vit, vitamin; CHD, congenital heart disease; NA, non-available. Bayley-III cognitive scores were interpreted as: normal, ≥ 85 points; mild, ≥ 70 and < 85 points; moderate, ≥ 55 and < 70 points; and severe, < 55 points (14, 15). NA, non-available. The sequence data of ALMS1 reference sequence NM_015120.4.

### Early- and late-onset in neonatal hypocalcemic seizures

Among 16 patients, the first seizures occurred were 5.3 ± 1.9 days after birth. Four had developed early-onset and 12 late-onset seizures. The mean age of first seizure in the early-onset group was 2.5 ± 0.6 days (range: 2-3 days), whereas that in the late-onset group was 6.2 ± 1.2 days (range: 5-8 days). In the early-onset group, the etiology was identified as HIE in two patients, vitamin D deficiency in one patient, and hypoparathyroidism in one patient.

### Different etiologies of neonatal hypocalcemic seizures

**Figure 1:**
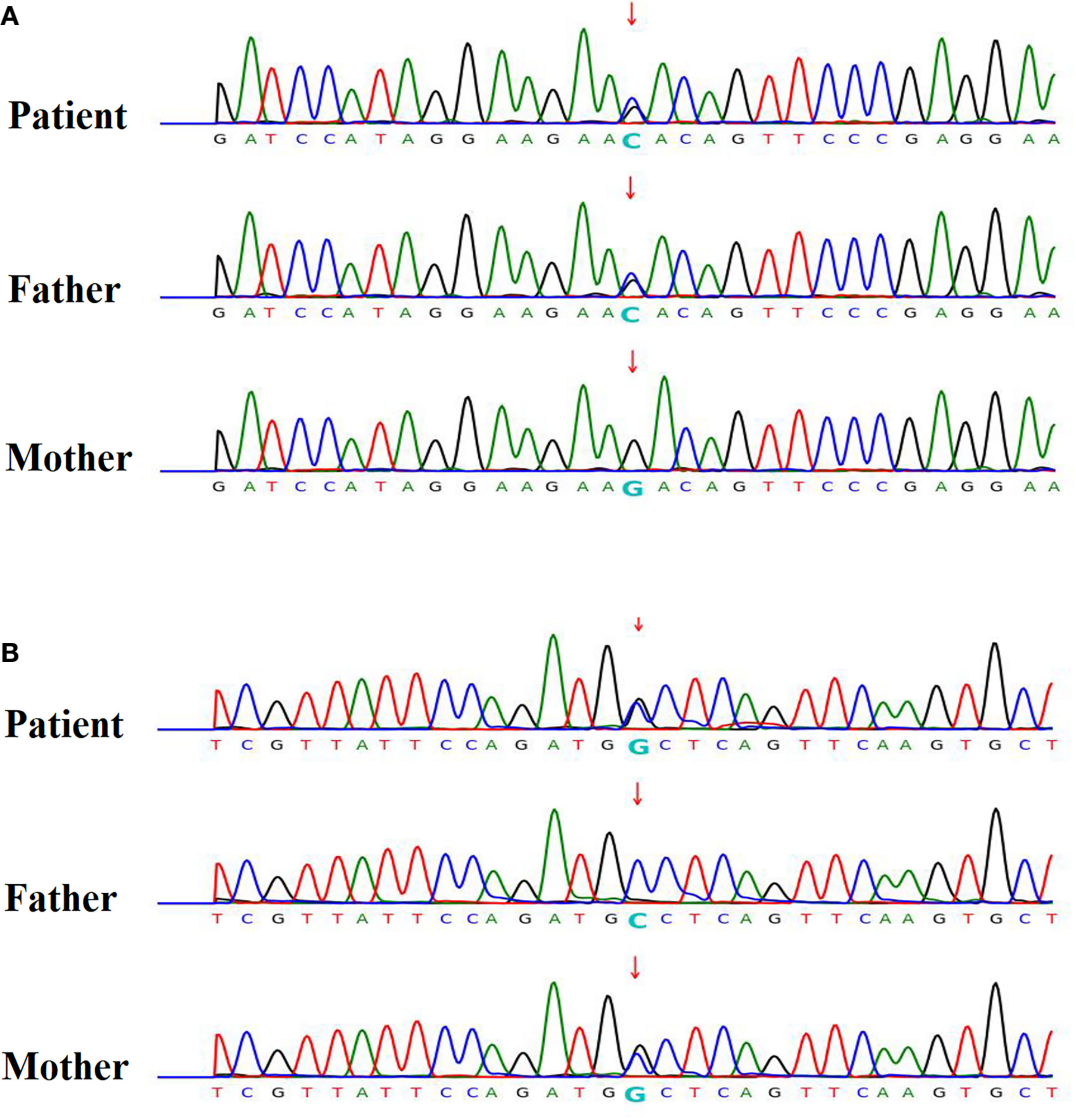
Patient 12 had hypocalcemic seizures which started to appear 8 days after birth in one newborn. The patient’s calcium level was 6.8 mg/dL (ion calcium, 0.71 mg/dL). The patient was diagnosed with Alström Syndrome with WES and was found to carry a compound heterozygous *ALMS1* (NM_015120.4) **(A)** c.10323G>C (p.K3441N) / **(B)** c.10079C>G (p.A3360G) mutation inherited from one each parent respectively. According to her phenotype, a Alström syndrome was diagnosed.

**Figure 2:**
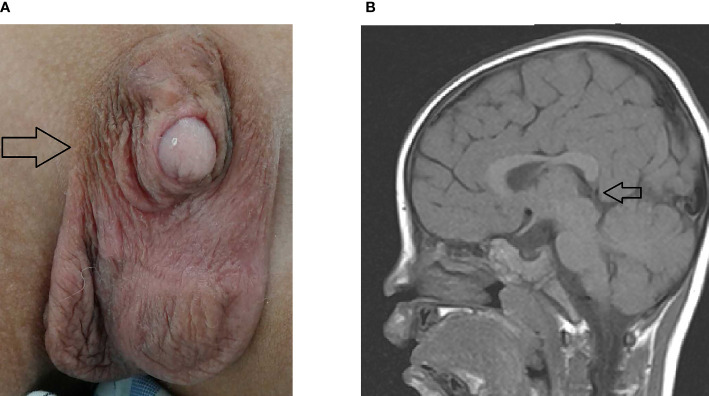
Patient 4 with hypocalcemic seizures that started 6 days after birth, had facial dysmorphism and hearing impairments. The patient’s calcium level was 6.0 mg/dL (ion calcium, 0.63 mg/dL). **(A)** He had ambiguous genitalia (arrow), scrotal bifida with hypospadias, penoscrotal transposition, congenital heart disease with double outlet right ventricle, ventricular septal defect, atrial septal defect, and pulmonary stenosis. Chromosome study confirmed the ring chromosome 9 and Kleefstra syndrome. **(B)** His MRI exhibited hypoplasia of splenium part of corpus callosum (arrow).

Five were diagnosed with syndromic neonatal HS and the etiologies included DiGeorge syndrome (three patients), Alström Syndrome (one patients), and Kleefstra syndrome (one patient). Of the 11 non-syndromic causes, eight patients were diagnosed with vitamin D deficiency, one with hypoparathyroidism, and two with HIE. All patients with syndromic hypocalcemic seizure had been identified a genetic disorder. Three patients with DiGeorge syndrome carried the 22q11.2 deletion and presented with neonatal HS combined with congenital heart disease. One neonatal HS carried compound heterozygous *ALMS1* gene mutation, which presented as Alström Syndrome. The Alström Syndrome carried compound heterozygous *ALMS1* ((NM_015120.4)) c.10079C>G (p.A3360G)/c.10323G>C (p.K3441N) mutations inherited from each parent (**Figure 1**). According to the ACMG guidelines for interpretation of genetic variants (21), the mutation of c.10079C>G (p.A3360G) in the *ALMS1* and the variant c.10323G>C (p.K3441N) were predicted to be “likely pathogenic” (PS2, PM2, PM3, PP3, and PP4). According to her phenotype and genetic study, a Alström Syndrome was diagnosed (22). She had 2 major criteria (compound heterozygous *ALMS1*mutations, nystagmus and dilated cardiomyopathy). One patient with Kleefstra syndrome carried a paired 9 ring chromosome at 3 months of age. His hypocalcemic seizures began 4 days after birth. He had facial dysmorphism, and hearing impairments. The patient’s initial calcium level was 6.0 mg/dL (calcium ion, 0.63 mg/dL). He also had ambiguous genitalia (**Figure 2A**), scrotal bifida with hypospadias, penoscrotal transposition, congenital heart disease with double-outlet right ventricle, ventricular septal defect, atrial septal defect, and pulmonary stenosis. Initially, phenobarbital and phenytoin were intravenously administered for the HS. His calcium levels returned to normal after 4 days of calcium supplementation; next, oral calcitriol was administered. His MRI revealed hypoplasia of the splenium part of the corpus callosum (**Figure 2B**). At the age of 2 years and 3 months, his seizures reappeared and were controlled by levetiracetam and pyridoxine. For the five syndromic neonatal HS, the mean seizure onset time in the syndromic neonatal HS group was 5.8 ± 1.3 days, was not different from those seizures in vitamin D deficiency group (5.9 ± 1.9) days.

### Vitamin D deficiency

All eight patients with vitamin D deficiency had 25-hydroxyvitamin D levels < 25 ng/mL, with an average of 17.5 ± 2.3 (range: 15 to 22) ng/mL. The mean seizure onset time was 5.9 ± 1.9 (range: 2 to 8) days. Four received only oral calcitriol treatment, and three with oral calcitriol and one antiseizure drug (intravenous phenobarbital). One patient (patient 11) need two antiseizure drugs (intravenous phenobarbital and phenytoin) due to refractory hypocalcemia and seizures. She had dilated cardiomyopathy and persistent pulmonary hypertension of newborn (PPHN) with respiratory failure. Her WES exhibited two *ALMS1c*.12118-65C>T and c.12118-65C>T (*in cis*). Her vitamin D level was 22 ng/mL, and was diagnosed vitamin D deficiency (**Table 1**).

### EEG and imaging

**Figure 3:**
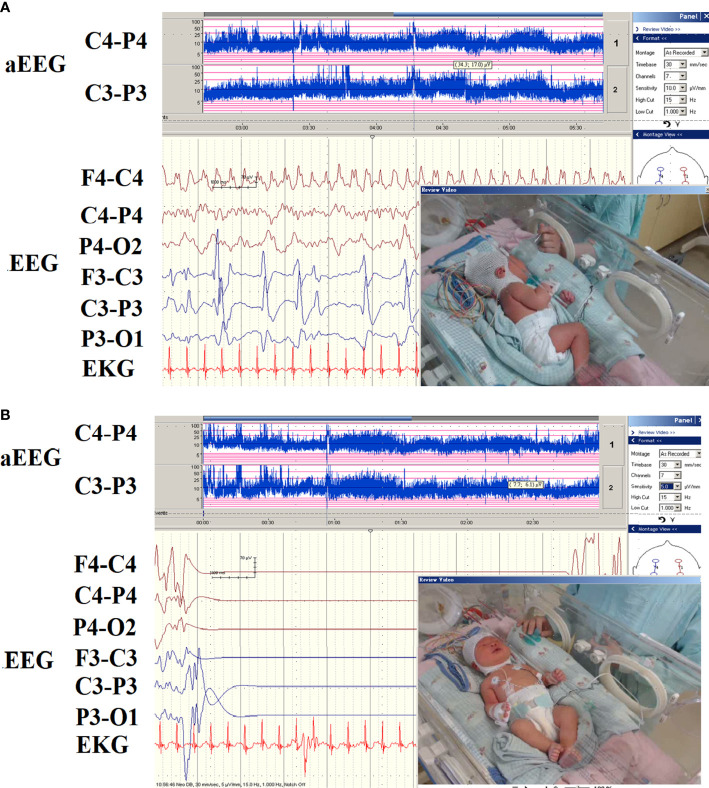
The EEG pattern of hypocalcemic seizures on the EEG monitor in patient 16. **(A)** The EEG monitor exhibited multifocal paroxysmal activity and **(B)** postictal inhibition of brain activity after seizure. The patients had seizures since day 6 of life due to hypocalcemia and vitamin D deficiency.

Out of 16 patients with EEG monitor, we were able to detect seizures by EEG monitoring in one patient (patient 16) only (**Figure 3**). The EEG initially showed the appearance of delta and theta spikes ictally, and multiple focal spikes interictally (**Figure 3A**). The EEG monitor exhibited the multifocal paroxysmal activity (**Figure 3A**) and postictal inhibition of brain activity after seizure (**Figure 3B**). For the findings of images. we detected six patients with magnetic resonance imaging (MRI) abnormalities, including 5 mild ventriculomegaly suspected of mild brain atrophy, and one patient with hypoplasia of splenium of corpus callosum in the Kleefstra syndrome patient (**Figure 2B**).

### Neurodevelopmental outcomes

Neurodevelopmental outcomes were evaluated after the age of 1 year. In the case of patients with vitamin D deficiency, all eight patients presented with unremarkable neurodevelopment outcomes. The neurodevelopment were worse in patients in the syndromic neonatal HS group than in those in the vitamin D deficiency group. In five syndromic neonatal HS, one exhibited severe delay, two moderate delay, one mild delay, and one unremarkable in the neurodevelopment. Two patients with HIE had unremarkable neurodevelopment at the age of 2 years.

## Discussion

An important contribution of this study is its delineation of the various etiologies in neonatal HS, including vitamin D deficiency, DiGeorge syndrome, and rare genetic syndromes, such as Kleefstra syndrome and Alström syndrome, which have previously never been reported as causes of neonatal HS. Our findings highlighted the complexity of syndromic neonatal HS, which can lead to a more refractory and complicated disease course. Patients in this group often do not respond well to anti-seizure drugs, and need to be managed by a multidisciplinary team comprised of different subspecialists. The diagnosis of syndromic neonatal HS often requires further genetic diagnostic methods, such as chromosome and WES study. Comorbidities in syndromic HS should be carefully managed to improve the outcomes.

Previously identified causes of neonatal HS from literature (**Table 2**) (3, 23–25). The etiologies are mostly vitamin D deficiency and DiGeorge syndrome. Sanjad et al. (25) reported a syndrome presenting with congenital hypoparathyroidism, severe intrauterine and postnatal growth retardation and several dysmorphic findings such as microcephaly, deep set eyes and thin lips in all patients, but without chromosomal anomalies and congenital heart disease, suggesting autosomal recessive inheritance resulting from parental consanguinity in the Arabian peninsula. Complex diseases in children, especially refractory seizures, have only recently been investigated using genetic testing, particularly WES. These genetic studies can predict the probable phenotypes and comorbidities, and aid the initialization of early treatment if available. However, the use of these methods has not been previously reported in studies of neonatal HS, which comprised various etiologies including nutritional deficiencies and genetic disorders. In this study, we identified DiGeorge syndrome and Alström Syndrome as one of the causes of neonatal HS. DiGeorge syndrome caused by a microdeletion on the long arm of chromosome 22 (22q11.2), is often associated with congenital heart disease. Hypocalcemia occurred in 69– 87% of the studies (26–28). Patients with DiGeorge syndrome present with specific facial features, frequent infections, developmental delay, learning problems, and cleft palate. Furthermore, Alström syndrome can also be diagnosed by WES and these patients present with progressive loss of vision and hearing, dilated cardiomyopathy (a form of heart disease that enlarges and weakens the heart muscle), obesity, type 2 diabetes, and short stature. This disorder can also cause serious and life-threatening medical problems involving several organ systems, including the kidneys, bladder, and lungs. HS in Alström syndrome have not been reported before. Due to advances in genetic studies, the phenotype and genotype of HS can be clarified, particularly for syndromic HS. The iPTH level was high in patient 12; HS can be due to secondary causes or other endocrine problems in Alström syndrome. The WES of patient 11 revealed two *ALMS1c*.12118-65C>T and c.12118-65C>T (*in cis)*; whether *in cis* mutations increased the risk of HS, and was refractory to calcium supply were unknown and requires further study. Kleefstra syndrome is presumed to be due to lower intake of milk due to feeding intolerance after birth and a comorbidity of congenital heart disease.

**Table 2:** Previously identified causes of neonatal hypocalcemic seizures from literature.

Authors	Methods and patients	Results	Significance	Reference
**Lynch et al., 1994**	15 neonatal HS	7 DiGeorge syndrome, 2 prematurity, 3 unknown, 1 jejunal atresia, 1 maternal hyperparathyroidism 1, and 1 idiopathic hypoparathyroidism	HS are rare but important treatable causes of neonatal seizures. The prognosis should be related to associated the diagnoses and etiology.	(3)
**Teaema et al., 2010**	19 symptomatic neonatal HS secondary to vitamin D deficiency	19 neonatal HS with 7 vitamin D deficiency (n = 13), stridor (n = 1) and jitteriness (n = 5). Their mean age of onset was 9.5 ± 7.8 days with a mean serum 25(OH)-D level of 9.4 ± 4.6 ng ml	Vitamin D supplementation should be considered after proper screening	(23)
**Levy-Shraga** **et al., 2015**	3 neonatal HS	1 Transient hypoparathyroidism due to maternal hyperparathyroidism, 1 vitamin D deficiency, and 1 DiGeorge syndrome.	Because many etiologies of neonatal HS, careful investigation of the specific etiology of the hypocalcemia is important.	(24)
**Sanjad et al., 1991**	12 infants with HS (6 boys, 6 girls); 9 neonatal HS	All had congenital hypoparathyroidism, growth failure, dysmorphism and mental retardation. None had chromosome anomalies. Eleven were born of parents who were intermarriage. None had CHD. Cell mediated immunity in 5 was normal. Four patients died. The remaining 8 were treated with vitamin D and calcium without change in their growth pattern.	A syndrome associated with HS and congenital hypoparathyroidism, suggested autosomal recessive inheritance from parental consanguinity in the Arabian peninsula.	(25)
**Huang et al.,** **2022**	16 neonatal HS	3 DiGeorge syndrome, 1 carried compound heterozygous *ALMS1* mutations with Alström syndrome, 1 Kleefstra syndrome, 8 vitamin D deficiency, 1 hypoparathyroidism, and 2 HIE	Syndromic neonatal HS can cause refractory HS, and need genetic study including WES	**In the study**

HS, hypocalcemic seizure; CHD, congenital heart disease; HIE, hypoxic-ischemic encephalopathies; WES, whole exome sequencing.

Many genetic or syndromic HS have heterogeneous presentations in the metabolic state and are associated with comorbidities (e.g., congenital heart disease). Alström syndrome is caused by an autosomal recessive of *ALDS* gene mutation. The metabolic problems in the disorder make it challenging for clinicians to manage the clinical problems associated with respiratory disorders and other endocrinological problems. The HS had a more benign course than other etiologies of seizures. We highlighted various etiologies of neonatal HS, including vitamin D deficiency, DiGeorge syndrome, and rare genetic syndromes (Kleefstra and Alström Syndrome), that were investigated by genetic studies. WES can be applied in children with refractory epilepsy or epileptic encephalopathy (29, 30). One study cohort consisted of 177 undiagnosed Japanese patients and yielded a 44% genetic diagnosis rate in children with complex diseases (31). However, for complex disorders, such as HS with other comorbidities, was not reported. Syndromic neonatal HS benefits from genetic testing and can cause refractory disease, which is often difficult to manage. Patients with vitamin D deficiency were found to have relatively better outcomes than those with syndromic HS.

Long-term EEG monitoring can detect seizures in high-risk newborns in critical care units. This method can aid in the rapid diagnosis of neonatal seizure and immediate initialization of effective treatment (32–35). Myoclonic seizures were found in 4 of the 16 cases. One patient had Digeorge syndrome, 2 had vitamin D deficiencies, and one had mild HIE. Hypocalcemia can cause myoclonic seizures, although not common (36, 37). This may be because the myoclonic seizure is brief and not persistent, similar to shock or tetany, which are not associated with epileptic seizures. Since some seizures are only visible in EEG recording (e.g., subclinical seizures) and their clinical manifestations may be subtle, many clinicians place increasing importance on EEG data to identify seizures in neonates (38). In neonatal HS, interictal EEG findings of newborns showed a relatively better background of EEG if the MRI is unremarkable. Focal spikes and paroxysmal activity were usually non-specific. Patients with neonatal seizure without brain structure anomalies had a different ictal EEG record from those with structural brain anomalies (33). In patients with seizures within the first two weeks after birth, the probable etiologies, including HIE, metabolic and genetic seizures, and HS, the ictal EEG from the EEG monitor can provide clues for the different etiologies earlier (33, 35, 39). In neonatal seizures caused by brain injury, ictal EEGs often demonstrate delta-theta waves during the seizure, whereas hypocalcemic or genetic seizures ever reported to be initially have unique fast activity that originates in one or both hemispheres (32–34). The characteristics of EEG findings can be helpful in determining whether the cause of seizure is genetic or non-genetic.

This study has some limitations, which can be attributed to several factors. Because of the rarity of neonatal HS, we presented a limited number of patients with different etiologies with a record of HS from a single medical center. Furthermore, the case-series presented included only hypocalcemic and symptomatic seizures, whereas subclinical seizures in neonatal hypocalcemia were underestimated. To detect the pattern of seizures associated with EEG is difficult in real word. As in the real world, it is not always possible to readily perform EEG to detect seizures and confirm their EEG correlations. As this was a retrospective study, it may have had a bias. However, using EEG monitors for HS can detect seizures, help understand seizure patterns, and provide clues for the etiologies and early treatment of seizures.

## Conclusion

Various etiologies of neonatal HS, including vitamin D deficiency, DiGeorge syndrome, and rare genetic syndromes (Kleefstra and Alström Syndrome) were investigated by genetic study. The findings revealed that syndromic neonatal HS benefit from genetic testing and can cause a refractory disease, which is difficult to manage. Patients with vitamin D deficiency were found to have a relatively better outcome than those with syndromic HS.

## Data availability statement

The datasets presented in this study can be found in online repositories. The names of the repository/repositories and accession number(s) can be found below: https://www.ncbi.nlm.nih.gov/, NM_015120.4.

## Ethics statement

The studies involving human participants were reviewed and approved by Chung Shan Medical University Hospital’s Internal Review Board (IRB #: CS14003). Written informed consent for participation was not provided by the participants’ legal guardians/next of kin because: This is a retrospective cases series. Written informed consent was not obtained from the minor(s)’ legal guardian/next of kin for the publication of any potentially identifiable images or data included in this article.

## Author contributions

I-CL conceptualized the research idea and devised the methodology. Y-CC and Y-CH collected the clinical data. I-CL drafted the manuscript and reviewed the manuscript. All authors contributed to the article and approved the submitted version.

## Funding

This work was supported by the Chung Shan Medical University Hospital grant FCU/CSMU 110-002 and MOST 111-2314-B-040 -028 -.

## Acknowledgments

We thank all those who participated in the present project.

## Conflict of interest

The authors declare that the research was conducted in the absence of any commercial or financial relationships that could be construed as a potential conflict of interest.

## Publisher’s note

All claims expressed in this article are solely those of the authors and do not necessarily represent those of their affiliated organizations, or those of the publisher, the editors and the reviewers. Any product that may be evaluated in this article, or claim that may be made by its manufacturer, is not guaranteed or endorsed by the publisher.
